# Risk Perception and Media in Shaping Protective Behaviors: Insights From the Early Phase of COVID-19 Italian Outbreak

**DOI:** 10.3389/fpsyg.2020.563426

**Published:** 2020-11-05

**Authors:** Benedetta Vai, Silvia Cazzetta, Davide Ghiglino, Lorenzo Parenti, Giacomo Saibene, Michelle Toti, Chiara Verga, Agnieszka Wykowska, Francesco Benedetti

**Affiliations:** ^1^Psychiatry and Clinical Psychobiology, Division of Neuroscience, IRCCS San Raffaele Scientific Institute, Milan, Italy; ^2^Vita-Salute San Raffaele University, Milan, Italy; ^3^Fondazione Centro San Raffaele, Milan, Italy; ^4^Istituto Italiano di Tecnologia, Genova, Italy; ^5^Quaestio Capital Sgr Spa, Milan, Italy; ^6^Department of Psychology, Sapienza University of Rome, Rome, Italy

**Keywords:** COVID-19, risk perception, media, social media, containment measures, protective behaviors, vaccine, efficacy

## Abstract

In the absence of target treatments or vaccination, the SARS-CoV-2 pandemic can be impeded by effectively implementing containment measures and behaviors. This relies on individuals’ adoption of protective behaviors, their perceived risk, and the use and trust of information sources. During a health emergency, receiving timely and accurate information enables individuals to take appropriate actions to protect themselves, shaping their risk perception. Italy was the first western country plagued by COVID-19 and one of the most affected in the early phase. During this period, we surveyed 2,223 Italians before the national lockdown. A quarter of the sample perceived COVID-19 less threatening than flu and would not vaccinate, if a vaccine was available. Besides, most people perceived containment measures, based on social distancing or wearing masks, not useful. This perceived utility was related to COVID-19 threat perception and efficacy beliefs. All these measures were associated with the use of media and their truthfulness: participants declared to mainly use the Internet, while health organizations’ websites were the most trusted. Although social networks were frequently used, they were rated lower for trustfulness. Our data differ from those obtained in other community samples, suggesting the relevance to explore changes across different countries and during the different phases of the pandemic. Understanding these phenomena, and how people access the media, may contribute to improve the efficacy of containment measures, tailoring specific policies and health communications.

## Introduction

The spread of the SARS-CoV-2 pandemic may have catastrophic consequences in terms of people’s well-being, welfare, and economic losses. In the absence of target medical treatments or vaccination, the pandemic can be impeded only by rapidly implementing protective behaviors (Betsch, 2020).

Many governments have activated unprecedented policies aimed at controlling the progress of the pandemic, while in a few countries, the implementation of these norms is still voluntary. In both cases, the effectiveness of containment measures depends on how the population perceives the risks associated with the contagion (Van Bavel et al., 2020).

In health psychology, the motivation to protect oneself from diseases is related to the perceived threat. As defined in the *protection motivation theory* (PMT), the perceived threat is derived from both how a person feels vulnerable to develop a certain condition and how severe it would be for him to be affected (Rogers, 1983; Floyd et al., 2000; Witte and Allen, 2000; Brug et al., 2009). Several studies confirmed significant, although small, relationships between the perceived vulnerability and severity with protective intentions and behaviors, including vaccinations (for a meta-analysis, see Brewer et al., 2007). Accordingly, the current COVID-19 risk perception may drive the adoption of protective behaviors. PMT also hypothesized that other relevant variables, such as efficacy beliefs, are key predictors of protective motivation (Rogers, 1983; Floyd et al., 2000; Witte and Allen, 2000). This dimension is usually defined as response efficacy (i.e., the perception of the effectiveness of the available protective actions in reducing the hazard) and self-efficacy (i.e., a person’s confidence on his ability to engage in such protective actions). Furthermore, risk perception is associated with information needs (Neuwirth et al., 2000). During a health emergency, receiving timely and accurate information enables individuals to take appropriate actions to protect themselves, in line with health agencies’ recommendations (World Health Organization, 2017). Health communications and interventions that increase risk appraisal and efficacy beliefs also lead to increase protective intentions and behaviors (Sheeran et al., 2014). Thus, to provide effective communication, understanding how a society uses and trusts different information sources (i.e., media) is of crucial relevance, considering their effect on perceived risk (Coleman, 1993; Reynolds and Seeger, 2005; Dudo et al., 2007; Lin and Lagoe, 2013; Kwok et al., 2020).

Assessing societal attitudes toward the current pandemic, in terms of people’s perceived risk, their attitudes toward containment measures and vaccines, along with their media use and trust, may have a large impact on pandemic management.

Previous insights on the early phase of the outbreak came from the Hong Kong and Vietnam communities (Huynh, 2020a; Kwok et al., 2020), where data indicate high levels of COVID-19 risk perception and adhesion to self-protective measures, as well as associations between these domains and usage of media. However, as the authors suggested, the previous experience of citizens with other epidemics, such as SARS, might have contributed to define “a secondary immune response” in terms of psychological and behavioral responses (Kwok et al., 2020).

Italy was the first western country plagued by COVID-19, and one of the most affected in the early pandemic. The first transmission was registered on 18 February 2020; 1 month later, positive cases increased to ∼47,000, revealing an exponential growth: differently from Hong Kong and Vietnam, Italy, as other western countries, did not have a recent “pandemic heritage.”

We analyzed risk perception, use and trust of media, and perceived utility of protective behaviors in 2,223 Italians recruited through an online survey in the first phase of outbreak, before the government legislated the lockdown in the whole country: 60% of our samples lived in Lombardia, the second Italian region for population density and the most affected one in that period.

## Methods

### Participants Recruitment

The survey was administered online from 27 February to 8 March 2020. The administration period covered an important phase for the pandemic in Italy: the first secondary transmission was registered on 18 February 2020, the first local emergency responses and quarantine measures were defined on 21 February (engaging two provinces for a total of 53,785 inhabitants), which culminated in lockdown measures in all the country on 8 March 2020 (around 60 million people). A software package, specifically developed for scientific online survey, was used to design the questionnaire (SoSci Survey, 2015^1^).

The study was advertised on authors’ contacts and their referrals and on different universities and city social groups through different social media (e.g., Facebook, Instagram, and WhatsApp). Participants were invited to complete the survey via a hyperlink and to disseminate the study, identifying a non-probability voluntary response sampling. Individuals who were aged 18 or above, understood Italian, and provided their informed consent may complete the survey. Participants were informed of the purpose of the study, and their participation was completely voluntary and anonymous. The study was approved by the local ethics committee (i.e., IRCCS San Raffaele Scientific Institute).

### Participants Characteristics

Participants were asked about their demographics: gender, age, marital status, years of instruction, educational qualification, study area, employment status, socioeconomic status, as well as whether they had undergone a flu vaccine and would vaccinate for SARS-CoV-2. At the time the online survey was conducted, the infection rates were different across the country: we asked the participants to indicate the region of birth, the domicile, the type of city they lived in (i.e., number of inhabitants), whether and where they had traveled abroad in the past 6 months as well as in Italy in the last 2 weeks.

### Risk Perception

Participants were required to report measures of risk perception for COVID-19 (De Zwart et al., 2009) and other five harmful conditions: flu, HIV, heart attack, car accident, and health consequences related to climate change. Following PMT, participants rated for each condition:

Severity [“How serious—on a scale from 1 to 10—would it be for you if you got (disease) in the next year?”];

Vulnerability [“How likely do you think it is that you will develop or contract a (disease) in the next year; very unlikely (1) to very likely (5)”];

For COVID-19 and flu, the following additional efficacy belief questions were included:

Response-efficacy [“To what extent do you think people can take effective actions to prevent getting COVID-19/flu in case of an outbreak”; not at all (1) to very much (4)];

Self-efficacy [“How confident are you that you can prevent getting COVID-19/flu in case of an outbreak”; not confident (1) to very confident (4)].

For each participant, administering order for harmful conditions was randomized. The perceived threat was defined as the product of severity and vulnerability (De Zwart et al., 2009). Assuming that risk perception could vary among participants according to different individual factors (e.g., age, health conditions, personal history of exposure to viral infections), in a similar way for COVID-19 and flu, we considered scores provided for flu as an intrasubject control condition: we thus defined the relative COVID-19 threat risk perception as the difference between COVID-19 and flu scores.

### Preventive Measures

Participants were asked to rate how much a set of containment measures (i.e., washing hands, limiting social interactions, avoiding crowded places, staying home, and using masks) were useful in preventing the spread of the virus in everyday life [Strongly disagree (1) to Strongly Agree (5)].

### Information and Media Exposure

Participants were asked to rate the usage of different sources of information [Never (1) to Always (5)], how much they trusted on the quality/veracity of the information provided on these sources [No trust (1) to total trust (5)], and how much media affected the usage of containment measures (i.e., social distancing, face masks, and washing hands) [Not at all (1) to Totally (5)].

### Statistical Analyses

Frequency and proportion were tabulated. Associations between age, gender, years of education, and COVID-19 risk perception and efficacy belief measures were explored through ANOVA and Pearson correlations. Logistic regression was performed entering willingness to vaccinate for SARS-CoV-2 (Yes vs. No, coded 0 1), if a vaccine was available, as a dependent variable, while age, gender, educational level, relative COVID-19 threat perception, and efficacy beliefs as predictors. Association between willingness to vaccinate and relative COVID-19 threat perception (similar/lower than flu vs. higher than flu) was explored with Pearson’s chi-squared test. Associations between continuous variables (i.e., age, years of education, perceived utility of containment, relative threat and efficacy beliefs for COVID-19, and use and trust on media) were assessed through Pearson correlations. One-way ANOVAs were performed exploring effects of willingness to vaccinate and relative COVID-19 threat (similar/lower than flu vs. higher than flu) on the perceived utility of containment, use, and trust on media, and *post hoc* pairwise comparisons for significant effects were Bonferroni corrected for multiple comparisons. Statistical significance was set at *p* < 0.05 in all the analyses, which were performed in STATA 14 (Stata Statistical Software: Release 14, College Station, TX, United States: StataCorp LP).

## Results

### Demographics

A total of 6,376 clicked the survey hyperlink; 3,170 gave their consent to participate in the study and were aged 18 or above. Subjects who did not currently live in Italy or did not answer questions related to perceived utility of containment measures, risk perception for COVID-19, and willingness to vaccinate were removed case wise. The final sample included 2,223 participants. Most of the participants were female (30.4% male, 675 respondents), of young age (mean age 36.4, SD ± 13.3), well-educated (32.7% of respondents had a master’s degree), workers (55.2%, 1,228 respondents had a full-time job) (Supplementary Table 1), lived in Lombardia (59.2%, 1,315) and in metropolis (32%, 711) (Supplementary Table 2). Our sample is younger, more educated, and with a higher representation of females than reference data for Italian population (Supplementary Table 1). Furthermore, most of the participants never got vaccinated for the flu (67.6%).

From the travel history of participants (Supplementary Table 3) emerged the majority (66.9%, 1,487) who did not travel abroad in the last 6 months; however, most of the participants had traveled around Italy for work and pleasure in the last 2 weeks (66.9%, 1,487).

### Risk Perception Measures

**FIGURE 1:**
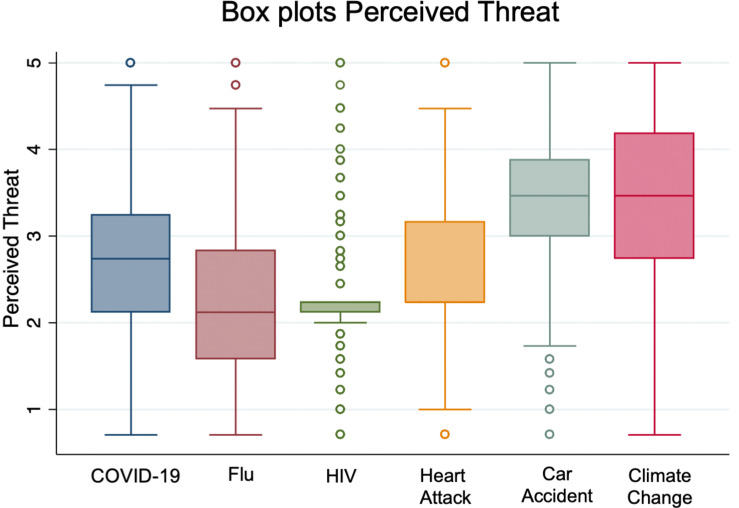
Perceived threat for evaluated harmful conditions (box plots).

Flu was rated as the least severe health condition, followed by COVID-19 (Table 1). On the contrary, flu was associated with the highest vulnerability, followed by consequences of climate change and, ranked third, COVID-19. Perceived threat was defined as the product of severity and vulnerability: flu had the lowest perceived threat compared to other conditions, COVID-19 was ranked third, after car accidents and climate change (Figure 1). For 46% of the participants, the probability of developing COVID-19 in the next year was perceived with a severity higher than 5 (subjects rated severity on a scale from 1 to 10), while only 19% for flu: ranking severity of COVID-19 higher than flu. However, 26% rated likely or very likely the probability to develop COVID-19 in the next year, against 41% for the flu (Supplementary Table 4). We also found that 24% of the sample perceived a higher threat related to flu than to COVID-19, while 13% considered them similar.

**TABLE 1:** Risk perception: mean and standard deviations.

**Harmful condition**	**Perceived severity**		**Perceived vulnerability**		**Perceived threat**	
	**Mean**	**SD**	**Mean**	**SD**	**Mean**	**SD**
COVID-19	5.36	2.47	2.95	0.98	2.70	0.87
Flu	3.50	2.14	3.28	1.15	2.25	0.87
Car accident	8.36	1.87	2.85	0.91	3.39	0.76
Climate change	7.62	2.29	3.08	1.19	3.36	1.04
Heart attack	8.75	1.97	1.74	0.85	2.66	0.75
HIV	8.89	1.99	1.33	0.65	2.36	0.61

*Perceived severity is the answer to “How serious (scale from 1 to 10) would it be for you if you got (the disease) in the next year?” Perceived vulnerability is the answer to “How likely do you think it is that you will develop or contract a (disease) in the next year; very unlikely (1) to very likely (5).”*

Females perceived COVID-19 threat higher than males (*F* = 11.7, η^2^ = 0.016, *p* < 0.001); however, no effects were found considering relative threat perception (COVID-19 vs. flu), suggesting no different effects between COVID-19 and flu risk perception. No significant associations with years of education and age were detected.

In terms of efficacy beliefs (Supplementary Table 5), most of the participants, respectively, 57.4 and 62.6%, were confident that people (response-efficacy) and themselves (self-efficacy) were able to prevent COVID-19. However, an oppositive trend can be observed for flu: only 38% indicated that people can prevent the disease and 48.6% referring to themselves. By performing paired *t*-tests on efficacy measures, results showed that self-efficacy was higher than response efficacy for both COVID-19 (*t* = 2.4, *d* = 0.05, *p* = 0.01) and flu (*t* = 8.9, *d* = 0.2, *p* < 0.001), indicating that participants considered themselves as being more effective in diseases protection than other people. However, both efficacy beliefs for COVID-19 were higher than those reported for flu (response efficacy: *t* = 19.2, *d* = 0.4; *p* < 0.001; self-efficacy: *t* = 13.3, *d* = 0.28, *p* < 0.001). Females reported a higher level of self-efficacy (*F* = 5.16, η^2^ = 0.006, *p* = 0.001), which was also positively associated to years of education (*r* = 0.08, *p* = 0.003). Both response- and self-efficacy were also directly related to age (*p* < 0.001, respectively, *r* = 0.1, *r* = 0.07).

### Preventive Measures

**FIGURE 2:**
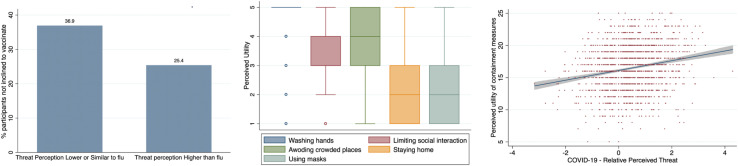
COVID-19 perceived threat and attitude to vaccination and containment measures: **(A)** COVID-19 threat perception and vaccination. **(B)** Perceived utility of containment measures. **(C)** Perceived utility of containment measures and threat perception.

Participants, 657 (29.5%), declared they would have not vaccinated for SARS-CoV-2, against 1,566 (70.4%) who would have vaccinated, if a vaccine had been available. Participants that perceived threat for COVID-19 as lower or similar than flu were more inclined to not vaccinate (Pearson χ^2^ = 32.5, *p* < 0.001) (Figure 2A).

The logistic regression showed significant predictors of the likelihood of vaccinating (*n* = 2,105; LR χ^2^ = 66.9; pseudo *R*^2^ = 0.02; *p* < 0.001). Specifically, the intention to not vaccinate was predicted by (a) lower relative COVID-19 perceived threat (*b* = −0.33, Std. Err = 0.05, *Z* = −5.96, *p* < 0.001, OR = 0.71), (b) lower response-effective (*b* = −0.2, Std. Err = 0.07, *Z* = −2.56, *p* = 0.011, OR = 0.82), (c) lower educational level (*b* = −0.11, Std. Err = 0.04, *Z* = −2.59, *p* = 0.009, OR = 0.89), and (d) higher age (*b* = 0.01, Std. Err = 0.004, *Z* = 3.39, *p* = 0.001, OR = 1.01). Self-efficacy, gender, and annual income did not exert significant effects. Participants also rated how much a set of protective behaviors (i.e., washing hands, limiting social interactions, avoiding crowded places, staying home, and using face masks) was perceived useful in preventing virus diffusion. Most of the participants agreed (also strongly) on the utility of washing hands and avoiding crowded places as measures to limit the spread of the virus, respectively, 94 and 74% (Figure 2B and Supplementary Table 6). For other protective behaviors, such as limiting social interactions, staying home, and using masks, the perceived utility was reduced, respectively, 45, 15, and 15%.

Higher perceived utility of containment measures was associated with higher relative perceived threat for COVID-19 (*r* = 0.2, *p* < 0.001) (Figure 2C) and higher levels of self- (*r* = 0.1, *p* < 0.001) and response efficacy (*r* = 0.13, *p* < 0.001). Those who perceived threat for COVID-19 as lower or similar to flu gave a lower rating to the utility of containment measures (η^2^ = 0.027; *p* < 0.001); this was confirmed for all the behaviors except for washing hands.

### Information and Media Exposure

**FIGURE 3:**
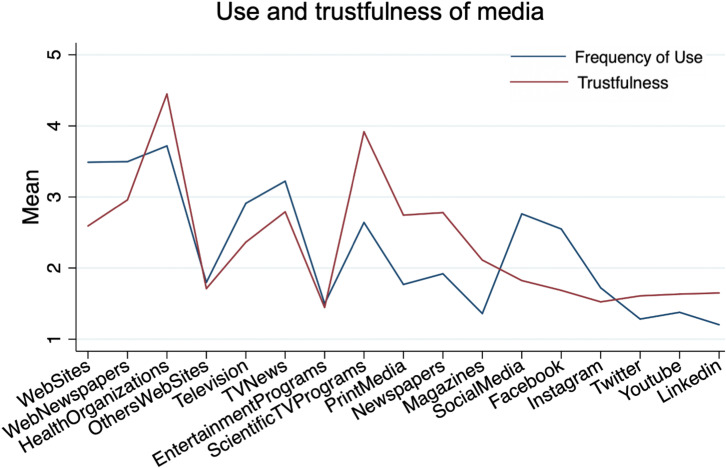
Use and trustfulness of media to keep informed about COVID-19.

Of the participants, 60% declared that they often/always consulted health organization websites (e.g., World Health Organization, Italian Ministry of Health) to keep informed on the current situation (Figure 3 and Supplementary Table 7). Websites in general and newspaper websites were also frequently consulted (∼51%), followed by TV news (45%). Only health organization websites were defined as trustable sources from most of the participants (86%). On the other hand, newspaper websites, TV news, and websites in general were rated trustful from, respectively, 27, 25, and 10% of the participants. Scientific TV programs were rated as good quality of information by 69%; however, they were frequently consulted only by 28%.

Printed media and printed newspapers were perceived as trusty media by ∼20% but, only ∼10% declared to often/always consented them. Social media were often/always consulted to keep informed by 30% of the participants; specifically, Facebook appeared the most used but less than 3% trusted information shared on them.

Higher use of media and higher rate of their trustfulness was related with a higher COVID-19 threat perception (use: *r* = 0.09, *p* < 0.001; trust: *r* = 0.07, *p* = 0.002), higher response-efficacy (use: *r* = 0.12, *p* < 0.001; trust: *r* = 0.1, *p* < 0.001) and self-efficacy (use: *r* = 0.06, *p* = 0.01; trust: *r* = 0.06, *p* < 0.01), and larger use of protective behaviors (use: *r* = 0.17, *p* < 0.001; trust: *r* = 0.19, *p* < 0.001). On the contrary, both those who were not inclined to vaccinate and who perceived threat for COVID-19 lower or similar to flu used less (attitude to vaccination: *F* = 14.7, η^2^ = 0.007, *p* < 0.001; perceived threat: *F* = 15.5, η^2^ = 0.008, *p* < 0.001) and trusted less media (attitude to vaccination: *F* = 22.5, η^2^ = 0.01, *p* < 0.001; perceived threat: *F* = 10.2, η^2^ = 0.005, *p* = 0.001). People who would have not vaccinated use less (*F* = 26.4, η^2^ = 0.01, *p* < 0.001) and trust less (*F* = 32, η^2^ = 0.01, *p* < 0.001) media, also institutional health originations’ website, which were generally more used in younger (*r* = −0.14, *p* < 0.001) and higher educated people (*r* = 0.08, *p* < 0.001). Accordingly, participants of both these groups reported a lower influence of media on the adherence to containment measures (attitude to vaccination: *F* = 89.3, η^2^ = 0.04, *p* < 0.001; perceived threat: *F* = 24.6; η^2^ = 0.01, *p* < 0.001).

## Discussion

According to our results, collected during the Italian first phase of the outbreak, a quarter of the surveyed Italians perceived COVID-19 less threatening than flu, and if a vaccine was available, they would not vaccinate. Most people perceived containment measures, based on social distancing and on wearing masks, as not useful. Attitude to vaccination and utility of protective behaviors were related to COVID-19 threat perception and efficacy beliefs. All these measures were associated with the use of media and their perceived truthfulness.

In more detail, 46% of the participants perceived being affected by COVID-19 as severe, but only 26% rated it as likely. Risk perception in Italy was strikingly lower compared to data obtained in the early phases of pandemic in Vietnam and Hong Kong (Huynh, 2020b; Kwok et al., 2020): in the latter, the corresponding percentages for severity and vulnerability were 97 and 89%. Furthermore, 37% of the participants perceived COVID-19 as a threat less or similar to flu, highlighting threat underestimation during the first phase of the outbreak. Females perceived COVID-19 as more threatening than males do, in line with recent evidence obtained in 10 plagued countries across Europe, America, and Asia (Dryhurst et al., 2020), consistent with higher reported risk perception in women (Kim et al., 2018). However, in our study, no differences were detected when results are compared to flu, suggesting no specific effect of gender specifically on COVID-19.

In line with previous meta-analysis (Sheeran et al., 2014) and recent worldwide findings on COVID-19 (De Bruin and Bennett, 2020; Dryhurst et al., 2020), a lower perceived threat was also associated with a lower perceived utility of containment measures. In our sample, most of the participants agreed on the utility of washing hands and avoiding crowded places as measures adopted in order to limit the spread of the virus, but other protective behaviors, such as limiting social personal interactions, staying home, and using masks, were perceived useful only from, respectively, 45, 15, and 15% of the participants. This might have contributed to the spread of the virus (Walter et al., 2012), resulting in an exponentially increase in cases in Italy during this first pandemic phase. An indirect comparison with Hong Kong data (Kwok et al., 2020) suggests that our responders perceived protective measures, e.g., wearing masks or social distancing, remarkably less useful.

Such evidence confirmed that perceived threat is a potential key factor in affecting positive containment measures, especially for social distancing norms. Notably, recent findings, which confirmed a significant association between risk perception and different containment measures during the early phase of the pandemic in the United States (10–12 March 2020), showed an increase of this relationship and levels of perceived risk and protective behaviors in a later stage (13–31 March 2020). These results suggest that measures related to risk perception may rapidly change paralleling the different pandemic phases. Our data have been collected before the national lockdown as soon after the first registered contagion, providing a cross section of the first approach to the virus in a western country.

Perceived efficacy identifies another relevant predictor of protective motivation (Rogers, 1983; Floyd et al., 2000; Witte and Allen, 2000): in our sample, higher COVID-19 response- and self-efficacy were related to a higher perceived utility of containment measures, as found in recent data on worldwide pandemic (Dryhurst et al., 2020; Mækelæ et al., 2020). Interestingly, our participants significantly reported both higher response- and self-efficacy for COVID-19 compared to flu (small to medium effect sizes) and rated themselves more efficient in preventing the diseases (self-efficacy) compared to other people (response-efficacy) for both the viruses, although for SARS-CoV-2, we detected a trivial effect (*d* = 0.05). These results may indicate an “optimistic bias,” i.e., the illusion of being less at risk than others from adverse events and illness, as previously found for COVID-19 (Dolinski et al., 2020) and in line with results detected in different countries comparing own to others’ efficacy (Mækelæ et al., 2020). From an overall perspective, most of the participants (∼60%) were confident that both themselves and other people can take effective actions to prevent COVID-19 in case of an outbreak. However, as previously highlighted, most containment measures, such as limiting social interactions, staying home, and using masks, were mainly perceived not useful in preventing the spread of the virus. Despite perceived efficacy is relevant in order to promote protective behaviors, efficacy beliefs should be accompanied by adequate knowledge of the correct prophylactic measures. Otherwise, unrealistic efficacy beliefs may result in a possible misleading “illusion of control,” i.e., tendency for people to overestimate their ability to control events (Langer, 1975), which may further expose people to increased risk of contagion. That is, contagions may increase exponentially, even if perceived efficacy is high, when risk perception and correct knowledge of prophylactic measures are low: in line with what we dramatically observed in Italy during this first period of pandemic. Combined with the “illusion of control,” an optimistic bias in probability estimates and information processing could explain why people estimate a higher efficacy for the more severe, and never experienced, COVID-19 over the less severe, and commonly experienced, flu, as well as for themselves than for others.

Our results suggested another crucial relationship: higher use of media and a higher rate of their trustfulness associated with higher COVID-19 threat perception, response- and self-efficacy, and use of protective behaviors, in line with previous findings (Huynh, 2020a). This relationship highlighted the crucial effect that media may exert in shaping risk perception and usage on protective behaviors. To collect information on COVID-19, participants declared to mainly use web sites of public health organizations (e.g., World Health Organization, Italian Ministry of Health), which also obtained the highest rate in terms of trustfulness, differently from the Hong Kong community, where only 16% of the respondents found information from official websites reliable or very reliable (Kwok et al., 2020). In our sample, scientific television programs and newspapers (both printed and in web format) received good ratings in terms of trustfulness. However, they were not frequently consulted, except for websites. Overall, the Internet was confirmed as the most used source. Although social networks were also quite consulted, they received a lower rate in terms of information quality. These results outlined a profound change compared to previous decades, when the Internet was significantly less used than other media (Walter et al., 2012).

The adherence to protective behaviors as well as vaccinations is extremely important in preventing epidemics (World Health Organization, 2020). Interestingly, risk underestimation has been demonstrated to reduce adhesion to containment measures and be a barrier to vaccination (Walter et al., 2012). Studies conducted on 2009 A/H1B1 virus or “swine flu” showed that the success of public health programs was largely dependent on individual risk perception: despite the vaccination was the most effective preventive intervention, only a low portion of the population got vaccinated (Renner and Reuter, 2012). Thus, to explore attitude to a SARS-CoV-2 vaccine may have a remarkable impact in tailoring the most effective health communication, preparing the population for its arrival. In our sample, around a third of our participants declared that they would not vaccinate for SARS-CoV-2, if a vaccine was available. This attitude was predicted by higher age, and lower relative COVID-19 perceived threat and response efficacy, in accordance with previous meta-analytic evidence for vaccinations (Brewer et al., 2007). These data suggested that specific health communication should be focused on vaccinations in the perspective of available vaccines for SARS-CoV-2 and that older people may particularly benefit from tailored media strategies, as defined as the at-risk population for COVID-19 disease. Notably, those who were not inclined to vaccinate used less media and judged the information less reliable, an effect detected also for institutional health originations’ websites. This kind of media was less used in older and less educated people. This indicates that media, or new media, should be shaped and tailored in order to achieve this part of the population, increasing their trust.

Although the detected relationships between the use of media, risk perception, and adoption of protective behaviors are small, in line with meta-analytic evidence (Brewer et al., 2007; Sheeran et al., 2014), we nevertheless support the necessity to incentivize people to refer to public health organizations and scientific sources also through other sources, such as television or social media. Media and social media should increase the broadcast of educational messages focused on personal hygiene, seeking early medication care and self-isolation. These measures would help in effectively limit the pandemic. However, risk communication should also be aimed at increasing both risk perception and efficacy beliefs, as our results suggest. On the one hand, fear, possibly related to a high-risk perception, induces changes in behavior only when subjects feel able to deal with threat (i.e., efficacy); on the other hand, fear may lead to defensive reactions such avoidance or reactance (Witte and Allen, 2000). Meta-analytic evidence showed that induced increase in risk appraisal had a larger impact in changing intentions and behaviors when either response and self-efficacy are simultaneously enhanced (Sheeran et al., 2014). Moreover, health communications should target vulnerable populations increasing adherence to correct protective behaviors, with specific attention to vaccines for the next future. Notably, relevant dissimilarities in terms of use and trust media may arise in different cultures or countries, as it appeared by comparing our results to some eastern countries’ data. Therefore, taking into account this variability may have a remarkable impact on defining the most effective health communication.

Our results confirmed previous insights concerning the role of risk perception and media in shaping protective behaviors. However, we have highlighted differences in the Italian population compared to other communities with a recent history of epidemics and a different trust and use of media during the early phase of a pandemic. We focused on a segment of the Italian population that lives in a geographical area with a high population density, deeply plagued by the virus, exactly during the beginning of the outbreak. Clearly, to explore how these findings change across different countries and during different phases of the pandemic may provide important insights on its management, together with its determinants and resulting behaviors. For example, previous studies highlighted that sociocultural variables, differently expressed in each country, can affect risk perception and the adoption of containment measure during COVID-19 pandemic (Dryhurst et al., 2020; Huynh, 2020c). Understanding these phenomena, and how people access to media, may contribute to improve the efficacy of containment measures, tailoring specific policies and health communications to target vulnerable populations and helping institutions worldwide. By highlighting the importance of media in influencing perceived threat and compliance to prophylactic measures, we implicitly suggest that public health policies should prompt the spread of sound scientific information though the Internet, as a foundation for a healthy world.

## Data Availability Statement

The datasets presented in this article are not readily available because of confidentiality and ethical restrictions. Requests to access the datasets should be directed to BV, vai.benedetta@hsr.it.

## Ethics Statement

The study involving human participants was reviewed and approved by the local ethical committe at IRCCS San Raffaele Scientific Institute. The participants provided their written informed consent to participate in this study.

## Author Contributions

All authors contributed to the conception and design of the study, data collection, and manuscript revision, and read and approved the submitted version. BV and SC organized the database. BV performed the statistical analysis and wrote the first draft of the manuscript. CV, LP, and GS contributed by writing sections of the manuscript.

## Conflict of Interest

The authors declare that the research was conducted in the absence of any commercial or financial relationships that could be construed as a potential conflict of interest.

## Footnotes

1 http://www.soscisurvey.de
