# Supplementary material for: Risk Perception and Media in Shaping Protective Behaviors: Insights From the Early Phase of COVID-19 Italian Outbreak
Source: Front Psychol. 2020 Nov 5;11:563426. doi: 10.3389/fpsyg.2020.563426 (PMC7674945; doi:10.3389/fpsyg.2020.563426)
Supplement: Supplementary file 1 [file Table_1.pdf]

Supplementary Material

1 Supplementary Tables

Supplementary Table 1. Study participants demographics and comparative data of Italian population (60.244.639 resident people). All data on Italian population is from the Italian National Institute of Statistics (ISTAT) at beginning of 2019; data on income levels is from Ministero dell'Economia e delle Finanze. Abbreviations: Ph.D., Doctor of Philosophy; N.A. Not available.

		Study Participants (N; %)		Italian population (%)
**Gender**	Male	675	30.4%	48.7%
	Female	1538	69.2%	51.3%
**Age**	18-24	325	14.6%	6.9%
	25-34	976	43.7%	10.8%
	35-44	357	16.1%	13.1%
	45-54	245	11,0%	16.1%
	55-64	241	10.8%	14.1%
	≥65	78	4.2%	21.9%
**Marital status**	Unmarried	1076	48.4%	42.66%
	Cohabitant	394	17.7%	N.A.
	Married	595	26.8%	47.21%
	Widowed/widower	12	0.5%	7.37%
	Separated	55	2.5%	N.A.
	Divorced	69	3.1%	2.76%
	Other	20	0.9%	N.A.
**Years of instruction**	5-8y	23	1.0%	N.A.
	9-13y	253	11.3%	N.A.
	14-16y	253	11.3%	N.A.
	17-18y	400	18.0%	N.A.
	19-21y	265	11.9%	N.A.
	≥22y	112	4.7%	N.A.
**Education qualification**	Primary school diploma	2	0.1%	16.2% [1]
	8th grade diploma	80	3.6%	32.4% [1]
	High-school diploma	717	32.3%	36.4% [1]
	Bachelor's degree	385	17.3%	15.0% [1, 2]
	Master's degree	728	32.7%	N.A.
	Specialisation degree	119	5.4%	N.A.
	PhD	186	8.4%	N.A.
**Study area**	Scientific	836	37.6%	N.A.
	Humanistic	778	35,0%	N.A.
	Diploma	360	16.2%	N.A.
	Other	221	9.9%	N.A.
**Employment status**	Full-time job	1228	55.2%	31.4%
	Part-time job	343	15.4%	7.4%
	Housewife	41	1.8%	13.2%

	Unemployed / Job searching	180	8.6%	4.3%
	Retired	72	3.2%	26.6%
	Stage	149	6.7%	N.A.
	Student	165	7.4%	17.1%
**Annual income**	<8.000 €/year	444	20,0%	23.9% [3]
	8.000-15.000 €/year	385	17.3%	20.0% [3]
	15.000-28.000 €/year	671	30.2%	35.3% [3]
	28.000-55.000 €/year	464	20.9%	16.2% [3]
	55.000-75.000 €/year	91	4.1%	2.2% [3]
	>75.000 €/year	56	2.5%	2.4% [3]
**Flu-vaccine**	Yes	712	32,0%	16.8%
	Not	1503	67.6%	83.2%

Notes: (1) of residents > 15y; (2) Bachelor's degree or more; (3) of total taxpayers (approx. 69% of pop.).

Supplementary Table 2 (A). Participants regions of birth and domicile.

Region	Birth		Domicile	
	PARTICIPANTS (N; %)		PARTICIPANTS (N; %)	
Abruzzo	21	0.9%	17	0.8%
Basilicata	13	0.6%	5	0.2%
Calabria	43	1.9%	12	0.5%
Campania	82	3.7%	44	2.0%
Emilia Romagna	79	3.6%	98	4.4%
Friuli-Venezia Giulia	25	1.1%	21	0.9%
Lazio	86	3.9%	100	4.5%
Liguria	138	6.2%	151	6.8%
Lombardia	1127	50.7%	1315	59.2%
Marche	23	1.0%	13	0.6%
Molise	7	0.3%	2	0.1%
Piemonte	100	4.5%	116	5.2%
Puglia	108	4.9%	56	2.5%
Sardegna	26	1.2%	11	0.5%
Sicilia	93	4.2%	44	2.0%
Toscana	100	4.5%	94	4.2%
Trentino-Alto Adige	27	1.2%	25	1.1%
Umbria	19	0.9%	11	0.5%
Valle D'Aosta	3	0.1%	2	0.1%
Veneto	81	3.6%	77	3.5%
Abroad	22	1.0%	N.A.	N.A.

Supplementary Table 2 (B). Type of city where participants lived.

TYPE OF CITY	PARTICIPANTS (N; %)	
A metropolitan city (more than a million inhabitants)	711	32.0%
A big city (between 500 thousand and one million inhabitants)	177	8.0%
A medium-sized city (between 250 thousand and 500 thousand inhabitants)	130	5.8%
A small town (between 50 thousand and 250 thousand inhabitants)	316	14.2%
A medium-sized country (between 10 thousand and 50 thousand inhabitants)	457	20.6%
A small town (between 1000 and 10 thousand inhabitants)	398	17.9%
A rural area (under 1000 inhabitants)	34	1.5%

Supplementary Table 3. Participants travels.

PREVIOUS TRAVELS		PARTICIPANTS (N; %)	
**Travel abroad in the last 6 months**	Yes	727	32.7%
	No	1487	66.9%
**If yes. where**	Europe	563	25.3%
	Asia	85	3.8%
	North America	49	2.2%
	South America	16	0.7%
	Oceania	5	0.2%
	Africa	42	1.9%
**Travel in Italy in the last 2 weeks**	Yes. for work	186	8.4%
	Yes. for pleasure	440	19.8%
	Yes. For work and pleasure	1487	66.9%
	No	108	4.9%

Supplementary Table 4. Risk perception.

HARMFUL CONDITION	PERCEIVED SEVERITY										PERCEIVED VULNERABILITY				
	**1**	**2**	**3**	**4**	**5**	**6**	**7**	**8**	**9**	**10**	**1**	**2**	**3**	**4**	**5**
**COVID-19**	112<br>5%	209<br>9.4%	293<br>13.2%	219<br>9.9%	358<br>16.1%	257<br>11.6%	280<br>12.6%	253<br>11.4%	101<br>4.5%	141<br>6.3%	168<br>7.6%	493<br>22.2%	975<br>43.9%	460<br>20.7%	127<br>5.5%
**Flu**	470<br>21.2%	426<br>19.2%	381<br>17.1%	226<br>10.2%	303<br>13.6%	190<br>8.5%	124<br>5.6%	63<br>2.8%	15<br>0.7%	25<br>1.1%	143<br>6.4%	403<br>18.1%	770<br>34.6%	503<br>22.6%	404<br>18.2%
**Car Accident**	18<br>0.8%	14<br>0.6%	31<br>1.4%	19<br>0.9%	125<br>5.6%	105<br>4.7%	237<br>10.7%	448<br>20.2%	368<br>16.6%	841<br>37.8	147<br>6.6%	554<br>24.9%	1099<br>49.2%	302<br>13.6%	104<br>4.7%
**Climate Change**	36<br>1.6%	51<br>2.3%	78<br>3.5%	69<br>3.1%	174<br>7.8%	168<br>7.6%	277<br>12.5%	448<br>20.2%	305<br>13.7%	607<br>27.3%	245<br>11%	445<br>20%	722<br>32.5%	499<br>22.4%	302<br>13.6%
**Heart attack**	38<br>1.7%	27<br>1.2%	25<br>1.1%	17<br>0.8%	77<br>3.5%	61<br>2.7%	107<br>4.8%	288<br>13%	396<br>17.8<br>%	1175<br>52.9%	1082<br>48.7	702<br>31.6%	366<br>16.5%	48<br>2.2%	13<br>0.6%
**HIV**	56<br>2.5%	23<br>1%	19<br>0.9%	10<br>0.4%	50<br>2.2%	42<br>1.9%	98<br>4.4%	255<br>11.5%	348<br>15.7%	1305<br>58.7%	1647<br>74.1%	417<br>18.8%	119<br>5.4%	14<br>0.6%	9<br>0.4%

Note: perceived severity is the answer to “How serious (scale from 1 to 10) would it be for you if you got [the disease] in the next year?”; Perceived vulnerability is the answer to “How likely do you think it is that you will develop or contract a [disease] in the next year; very unlikely (1) to very likely (5).

Supplementary Table 5. Efficacy beliefs.

HARMFUL CONDITION	RESPONSE EFFICACY						SELF-EFFICACY					
	**mean**	**SD**	**1**	**2**	**3**	**4**	**mean**	**SD**	**1**	**2**	**3**	**4**
**COVID-19**	2.64	0.69	72<br>3.2%	874<br>39.32%	1.069<br>48.9%	208<br>9.26%	2.67	0.64	48<br>2.2%	793<br>35.6%	1222<br>54.9%	162<br>7.24%
**Flu**	2.33	0.7	199<br>8.9%	1179<br>53%	749<br>33.7%	96 4.3%	2.47	0.68	132<br>5.9%	1011<br>45.5%	971<br>43.7%	109<br>4.9%

Note: Response-efficacy is the answer to "To what extent do you think people can take effective actions to prevent getting COVID-19/flu in case of an outbreak"; not at all (1) to very much (4); Self-efficacy is the answer to "How confident are you that you can prevent getting COVID-19/flu in case of an outbreak"; not confident (1) to very confident (4).

Supplementary Table 6. Perceived Utility of different protective behaviors in preventing virus spreading.

**BEHAVIOURS**	**PERCEIVED UTILITY**						
	**mean**	**SD**	**1 Strongly Disagree**	**2 Disagree**	**3 Neither agree nor disagree**	**4 Agree**	**5 Strongly Agree**
**Washing your hands often**	4.72	0.67	31<br>1.4%	19<br>0.9%	38<br>1.7%	362<br>16.3%	1773<br>79.8%
**Limiting your interaction with others**	3.30	1.08	115<br>5.2%	439<br>19.7%	620<br>27.9%	769<br>34.6%	280<br>12.6%
**Avoiding crowded places**	3.93	0.97	47<br>2.1%	165<br>7.4%	353<br>15.9%	990<br>44.5%	668<br>30%
**Staying home**	2.20	1.11	730<br>32.8%	723<br>32.5%	443<br>19.9%	258<br>11.6%	69<br>3.1%
**Wearing face-mask protection**	2.32	1.06	558<br>25.2%	771<br>34.8%	574<br>25.9%	250<br>11.3%	62<br>2.8%

Supplementary Table 7. Media usage and trustfulness.

**MEDIA**		**USAGE**							**TRUSTFULNESS**						
		**mean**	**SD**	**1 Never**	**2 Rarely**	**3 Some times**	**4 Often**	**5 Always**	**mean**	**SD**	**1 No Trust**	**2**	**3**	**4**	**5 Total Trust**
**Websites**	Websites (generic)	3.49	1.35	238<br>10.7%	286<br>12.9%	446<br>20.1%	495<br>22.3%	650<br>29.2%	2.59	0.84	212<br>9.5%	688<br>30.9%	978<br>44%	209<br>9.4%	21<br>0.9%
	web newspapers	3.50	1.33	254<br>11.4%	237<br>10.7%	453<br>20.4%	554<br>24.9%	621<br>27.9%	2.96	0.94	152<br>6.83%	463<br>20.8%	900<br>40.5%	538<br>24.2%	71<br>3.2%
	health organizations	3.72	1.31	200<br>9%	212<br>9.5%	358<br>16.1%	556<br>25%	789<br>35.5%	4.45	0.76	18<br>0.8%	33<br>1.5%	147<br>6.6%	703<br>31.6%	1221<br>54.9%
	other / blog	1.80	1.12	1194<br>53.7%	424<br>19.1%	274<br>12.3%	122<br>5.5%	84<br>3.8%	1.71	0.85	1065<br>47.9%	668<br>30%	300<br>13.5%	63<br>2.8%	10<br>0.5%
**TV**	TV (generic)	2.91	1.41	470<br>21.1%	385<br>17.3%	457<br>20.6%	399<br>17.9%	370<br>16.6%	2.36	0.96	435<br>19.6%	723<br>32.5%	707<br>31.8%	200<br>9%	30<br>1.4%
	news	3.22	1.42	363<br>16.3%	330<br>14.8%	429<br>19.3%	476<br>21.4%	526<br>23.7%	2.79	1.08	283<br>12.7%	551<br>24.8%	720<br>32.4%	463<br>20.9%	104<br>4.7%
	entertainment	1.50	0.95	1524<br>68.6%	297<br>13.4%	165<br>7.4%	77<br>3.5%	49<br>2.2%	1.45	0.77	1466<br>65.9%	418<br>18.8%	176<br>7.9%	48<br>2.2%	7<br>0.3%
	scientific	2.64	1.38	654<br>29.4%	332<br>14.9%	496<br>22.3%	391<br>17.6%	244<br>11%	3.92	0.96	57<br>2.6%	95<br>4.3%	436<br>19.6%	911<br>41%	623<br>28%
**Printed media**	Printed media (generic)	1.77	1.12	1260<br>56.7%	350<br>15.7%	271<br>12.2%	153<br>6.9%	66<br>3%	2.74	0.96	257<br>11.6%	489<br>22%	939<br>42.2%	362<br>16.3%	51<br>2.3%
	newspapers	1.92	1.22	1168<br>52.5%	352<br>15.8%	312<br>14%	179<br>8%	109<br>4.9%	2.78	1.03	282<br>12.7%	478<br>21.5%	860<br>38.7%	424<br>19.1%	75<br>3.4%

	magazines	1.36	0.78	1633<br>73.5%	276<br>12.4%	133<br>6%	43<br>1.9%	22<br>1%	2.11	0.98	693<br>31.2%	669<br>30.1%	580<br>26.1%	144<br>6.5%	20<br>0.9%
**Social media**	Social media (generic)	2.76	1.32	495<br>22.3%	407<br>18.3%	562<br>25.3%	394<br>17.7%	250<br>11.2%	1.82	0.84	899<br>40.4%	747<br>33.6%	404<br>18.2%	57<br>2.6%	3<br>0.1%
	Facebook	2.55	1.40	703<br>31.6%	394<br>17.7%	443<br>19.9%	321<br>14.4%	261<br>11.7%	1.69	0.81	1071<br>48.2%	696<br>31.3%	309<br>13.9%	42<br>1.9%	4<br>0.2%
	Instagram	1.72	1.19	1401<br>63%	262<br>11.8%	208<br>9.4%	124<br>5.6%	119<br>5.4%	1.52	0.76	1310<br>58.9%	528<br>23.8%	237<br>10.7%	32<br>1.4%	2<br>0.1%
	Twitter	1.28	0.81	1828<br>82.2%	106<br>4.8%	84<br>3.8%	61<br>2.7%	35<br>1.6%	1.61	0.86	1262<br>56.8%	470<br>21.1%	304<br>13.8%	59<br>2.7%	6<br>0.3%
	YouTube	1.38	0.87	1691<br>76.1%	205<br>9.2%	122<br>5.5%	60<br>2.7%	43<br>1.9%	1.63	0.83	1193<br>53.7%	524<br>23.6%	330<br>14.9%	42<br>1.9%	4<br>0.2%
	LinkedIn	1.20	0.65	1874<br>84.3%	120<br>5.4%	73<br>3.3%	28<br>1.3%	20<br>0.9%	1.65	0.90	1243<br>55.9%	422<br>19%	355<br>16%	61<br>2.7%	11<br>0.5%
